# The effects of temporary staff on observable teamwork outcomes within operating rooms

**DOI:** 10.3389/frhs.2025.1514431

**Published:** 2025-06-10

**Authors:** Logan Butler, Andrew Kozlow, Cody Mitchell, Rebecca S. Cintron, Caprice Greenberg, Lawrence B. Marks, Jin H. Ra, Lukasz Mazur

**Affiliations:** ^1^ Division of Healthcare Engineering, School of Medicine, University of North Carolina at Chapel Hill, Chapel Hill, NC, United States; ^2^ Department of Surgery, School of Medicine, University of North Carolina at Chapel Hill, Chapel Hill, NC, United States; ^3^ Department of Radiation Oncology, School of Medicine, University of North Carolina at Chapel Hill, Chapel Hill, NC, United States; ^4^ UNC Health, University of North Carolina at Chapel Hill, Chapel Hill, NC, United States; ^5^ Division of Acute Care Surgery, Department of Surgery, School of Medicine, University of North Carolina at Chapel Hill, Chapel Hill, NC, United States; ^6^ School of Information and Library Science, University of North Carolina at Chapel Hill, Chapel Hill, NC, United States

**Keywords:** teamwork observations, surgery, temporary staff, TeamSTEPPS, communication

## Abstract

**Introduction:**

The use of temporary nursing and operating room staff has increased, particularly following the COVID-19 pandemic. Despite this, limited research examines how temporary staffing impacts communication and teamwork behaviors in the operating room.

**Materials and methods:**

Surgical case safety behaviors were assessed using the Teamwork Evaluation of Non-Technical Skills (TENTS) tool at a large academic hospital. Mean scores for the 20 TENTS metrics were calculated for cases involving temporary staff and compared to cases with only permanent staff using two-sample *t*-tests. An analysis of variance (ANOVA) was also conducted to compare three staff compositions: only permanent staff, either a temporary scrub technician or circulating nurse, both a temporary scrub technician and circulating nurse.

**Results:**

Data from 100 surgical cases indicated that all TENTS safety behavior scores averaged above 2, suggesting acceptable behavior levels. Statistical analyses revealed no significant differences (*p* > 0.05) between TENTS scores in cases with temporary staff (*N* = 47) and permanent staff (*N* = 53). ANOVA showed no significant differences (*p* > 0.05) in TENTS metrics among the different staff compositions.

**Discussion:**

Standardized evaluations of teamwork behaviors revealed no significant differences between cases involving permanent and temporary operating room staff. This suggests that teams maintain communication and safety standards regardless of staff composition, potentially due to effective leadership, standardized protocols, and a strong institutional safety culture. Further research must explore the long-term effects of temporary staffing on patient safety and clinical outcomes, and to identify best practices for integrating temporary personnel into surgical teams.

## Introduction

The shortage of healthcare workers (HCWs) has been a persistent issue, exacerbated by the COVID-19 pandemic. Between February 2020 and November 2021, nearly 500,000 HCWs left their positions (1) Consequently, the use of traveling nurses in the United States surged by 35% in 2020 compared to 2019, with projections indicating a further 40% increase in the subsequent years (2). As the reliance on temporary staff grows, understanding how these workforce changes impact healthcare team communication, patient safety and other key outcomes has become a crucial area of interest.

Prior to the pandemic, several studies explored the effects of temporary staff on clinical outcomes. One study found higher rates of burnout, medication errors, patient complaints, and incidents of verbal abuse in hospitals with higher proportions of temporary staff, even after adjusting for nurse characteristics and staffing levels (3). However, these trends were often observed in resource-limited hospitals, which may contribute to the variations in patient outcomes (3). Another investigation revealed that essential care tasks were more likely to be missed in departments with increased temporary staffing (4). Interestingly, severely understaffed units with only permanent staff missed fewer tasks than fully staffed units with high levels of temporary workers (4).

Previous research has also indicated that temporary staff may distract permanent employees, complicating the workflow and increasing the burden on permanent team members (5). Additionally, patients exposed to care by temporary staff for more than 1.5 h from registered nurses (RNs) and 0.5 h from nursing assistants (NAs) during admission face a higher risk of mortality for each day of elevated temporary care (6).

The underlying reasons for these disparities are likely multifactorial. In surgical settings, studies have identified breakdowns in communication and teamwork as major contributors to clinical errors (7, 8). When the root causes of errors are examined, minor miscommunications and teamwork lapses accumulate and can be just as detrimental as single, significant human errors (9, 10). At an organizational level, factors such as staff behavior, variability in case demands, technical competence, and shared perceptions of ideal teamwork significantly impact team dynamics and outcomes (11). The integration of temporary staff into complex surgical teams, each with unique institutional policies and local cultures, introduces potential risks. In these environments, lapses in communication and an increased likelihood of clinical errors may occur.

This study aimed to utilize a valid evaluation method to quantify the communication behaviors of operating room (OR) teams and assess whether differences exist when temporary staff are present compared to when only permanent staff are involved. Furthermore, the study sought to determine if these differences varied based on the specific roles of team members within the OR.

## Material and methods

This study was conducted at a large academic medical center and received approval from the Institutional Review Board (IRB). Surgical cases were observed and evaluated using the Teamwork Evaluation of Non-Technical Skills (TENTS) tool as part of a cross-sectional observational study. Statistical analyses were conducted to compare mean TENTS scores using two-sample *t*-tests and ANOVA for cases involving various combinations of temporary and permanent surgical staff.

### TENTS observations

**Figure 1:**
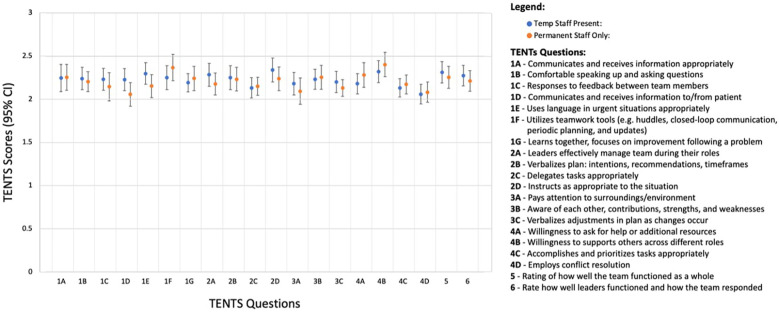
TENTS scores—temporary vs. permanent staff present during surgery.

TENTS tool was employed to assess safety behaviors during 100 surgical cases. Observations were carried out between November 2022 and April 2023. The primary outcome was the mean score for each TENTS item (Figure 1, right side of the chart) (12). The TENTS tool is derived from the Agency for Healthcare Research Quality Team Strategies and Tools to Enhance Performance and Patient Safety (TeamSTEPPS) training curriculum, which evaluates four essential teamwork behavior categories: communication, leadership, situation monitoring, and mutual support (13). The original scoring system was adapted from a 0-to-4 scale to a 0-to-3 scale to accommodate evaluations performed by medical students, where 0 = Behavior Not Observed, 1 = Poor Behavior, 2 = Average Behavior, and 3 = Excellent Behavior.

Seventeen first- and second-year medical students, after completing 1.5 h of TeamSTEPPS/TENTS scoring training, conducted the observations. Students were assigned to observe surgical cases that matched their scheduling availability. In accordance with IRB protocol, verbal consent was obtained from all surgical personnel prior to the commencement of TENTS observations. Additionally, demographic data of the surgical staff involved in the observed cases were collected for analysis.

### Statistical analysis

TENTS scores for each question were compared using a two-sample *t*-test between cases involving only permanent staff (*N* = 53) and cases involving at least one temporary staff member (*N* = 47). ANOVA was also used to assess differences in TENTS scores across three conditions: cases involving only permanent staff, cases with one temporary scrub technician or circulating nurse, and cases with both a temporary scrub technician and circulating nurse. Furthermore, ANOVA was employed to examine whether case duration—a proxy for case complexity—had any effect on teamwork behavior scores. All statistical analyses were performed using an alpha level of 0.05, with two-tailed tests. Our study was powered at 80% to detect relatively small effect size of 0.2 change in teamwork behaviors given 0.5 standard deviation (14).

## Results

Demographic data regarding personnel, staff roles, department, and case information during the TENTS tool observations are summarized in Table 1. The direct observations of teamwork behaviors across all OR cases using the TENTS tool are displayed in Figure 1, with all behaviors scoring above 2, indicating acceptable performance levels.

**Table 1:** Demographic information for collected observations.

Participants:	*N*	%	Cases:	*N*	%
Attendings:					
Male	23	65.7		79	78.2
Female	12	34.3		24	23.8
Residents:					
Male	21	51.2		43	42.6
Female	20	48.8		52	51.5
Fellows:					
Male	4	57.1		16	15.8
Female	3	42.9		8	7.9
Departments:	Cases (*N*)	Cases (%)			
Vascular	23	22.8			
ACS	21	20.8			
Surgical Oncology	19	18.8			
Orthopedics	11	10.9			
MIS/GI	10	9.9			
Cardiothoracic	9	8.9			
ENT	3	3.0			
Plastics	2	2.0			
Gyn/Onc	2	2.0			
Neurosurgery	1	1.0			
Type of Staff Present:	Cases (*N*)	Cases (%)			
Permanent Staff Only	53	52.5			
Temporary (Circulating Nurse)	14	13.9			
Temporary (Scrub Technician)	18	18.8			
Temporary (Both Circulatory and Scrub)	15	14.9			
Case Category:	Cases (*N*)	Cases (%)			
Elective	92	91.1			
Emergent	9	8.9			
Average Case Length:					
H:MM	2:52				

Statistical analysis revealed no significant differences in any TENTS question scores between cases involving temporary staff (*N* = 47) and those with exclusively permanent staff (*N* = 53) (all *p*-values > 0.05). Further analysis using ANOVA to compare cases with only one temporary staff member present (*N* = 32) (either a temporary scrub technician or circulating nurse), both a temporary scrub technician and circulating nurse present (*N* = 15), and cases with exclusively permanent staff (*N* = 53) also showed no significant differences in any TENTS metrics (all *p*-values > 0.05).

Additional analysis identified that the duration of the surgical case was negatively associated with several specific teamwork behaviors. Significant declines in TENTS scores were observed for the following questions: 1C—Response to Feedback (*p* < 0.05), 1E—Uses Language in Urgent Situations Appropriately (*p* < 0.01), 1G—Learns Together, Focuses on Improvement Following a Problem (*p* < 0.01), 3A—Pays Attention to Surroundings/Environment (*p* < 0.05), and 4D—Employs Conflict Resolution (*p* < 0.01).

## Discussion

Previous research has raised concerns about the impact of temporary nursing staff, with studies reporting an increase in care left undone (4), distractions for permanent staff (5), and higher risks to patients receiving care from temporary nurses (6). Other studies have suggested that breakdowns in communication and team behaviors lead to worse clinical outcomes including major complications or death (7). To our knowledge, this study is the first to assess and compare teamwork behaviors of both permanent and temporary staff in the OR using a standardized methodology. Despite the worrisome findings in earlier literature, our observations using the TENTS tool did not reveal any significant differences in behaviors between cases involving temporary and permanent staff. This suggests that surgical teams, regardless of staffing composition, were able to effectively utilize teamwork, maintain situational awareness, and employ clear communication and leadership to support each other and ensure patient safety. This positive outcome may reflect the institution's effective leadership, standardized teamwork and communication protocols, and a strong safety culture. The importance of effective leadership and chain of command, as well as clear protocols, have previously been identified as instrumental factors to ensure patient safety (15).

Our analysis did reveal a negative correlation between the duration of surgery and certain behaviors, such as response to feedback, appropriate use of language in urgent situations, learning from problems, situational awareness, and conflict resolution. Longer surgeries are inherently more complex and involve more handoffs, which could explain these findings. Previous research has also linked longer surgeries with increased risks of complications, emphasizing the importance of optimizing workflow and operative efficiency to mitigate such risks (16). While further study is needed to explore the relationship between case duration and teamwork behaviors in more detail, our findings suggest that temporary staffing does not exacerbate these issues.

The reassuring aspect of our study is that temporary staff did not negatively impact observed communication behaviors in the OR, contrary to the implication of earlier studies. Several factors may explain this. At the institution where the study took place, temporary staff undergo rigorous vetting processes to ensure they meet the necessary qualifications, and they receive training that facilitates communication and integration into the team. Additionally, temporary staff often bring diverse experiences from other institutions, which may help them adapt to new environments more effectively. Operating room staff at the institution where the study took place primarily work within the specialties in which they were observed, which expands their expertise on the cases in which they are participating in. Permanent staff's leadership, expertise in the field, and mentorship could also play a key role in supporting temporary staff and promoting smooth teamwork in the OR.

Our findings, as quantified using the TENTS tool suggest that team communication behaviors in the OR remain at an acceptable level, regardless of staffing composition. However, there is a possibility that TENTS tool was insensitive or has a ceiling effect. We selected the TENTS instrument over alternatives (e.g., TeamSTEPPS Team Performance Observation Tool) because we needed a more concentrated assessment of nontechnical teamwork elements. TENTS is specifically tailored to evaluate nontechnical aspects of teamwork, including interactions among team members, task allocation, and information management, making it especially pertinent in contexts where technical abilities are not the main focus. Furthermore, TENTS offers a more thorough observational framework, facilitating an in-depth analysis of particular teamwork behaviors, unlike the broader categories addressed by the other tools.Future work should explore the potential benefits of improving teamwork from acceptable to exceptional levels and investigate whether optimizing staffing models or enhancing safety behaviors has a more significant impact on clinical outcomes. Furthermore, more research is needed to correlate direct observations of teamwork behaviors with postoperative patient outcomes to fully understand the clinical implications of staffing practices.

There are several limitations to our study that affect its generalizability. First, OR staff were aware they were being observed, and verbal consent was required from all individuals present. This may have introduced bias, as those more comfortable with observation might have demonstrated more positive behaviors than those who opted out. Second, observations were performed by first- and second-year medical students. Though the observers received extensive standardized training, their limited medical training and expertise could lead to variability in scoring. Additionally, assessments of procedures are still subject to personal interpretation by the observers. An attempt to mitigate this inherent variability was made through rigorous training, but limits to the internal validity of the study remain. To enhance the generalizability of these findings, future studies should conduct additional clinical observations across different institutions and departments.

## Data Availability

The original contributions presented in the study are included in the article/Supplementary Material, further inquiries can be directed to the corresponding author.
